# Personalized Profiles of Autonomic Regulation in Elite Athletes: Analysis of Genetic and Cardiorespiratory Determinants Using Decision Tree Modeling

**DOI:** 10.3390/jpm16040230

**Published:** 2026-04-21

**Authors:** Irina Bacheva, Lyazat Ibrayeva, Dina Rybalkina, Irina Kadyrova, Diana Zhumagaliyeva

**Affiliations:** 1 Department of Internal Medicine, Karaganda Medical University, Karaganda 100012, Kazakhstan; bacheva@qmu.kz (I.B.); libraeva@qmu.kz (L.I.); rybalkina@qmu.kz (D.R.); 2 Institute of Life Sciences, Karaganda Medical University, Karaganda 100012, Kazakhstan; ikadyrova@qmu.kz

**Keywords:** sports medicine, personalized medicine, genes, autonomic nervous system, respiratory function, athletes

## Abstract

**Backgrounds**: The aim of this pilot study was to evaluate the hierarchical contribution of individual genetic polymorphisms to the variability of autonomic regulation parameters and respiratory function in athletes of different sport specializations using Classification and Regression Tree (CRT) analysis. **Methods**: The study included athletes divided into two groups: hockey players (*n* = 48) and martial artists (*n* = 43). Heart rate variability (LF, HF) parameters and spirometric indices (FEV_1_) were assessed. Genetic analysis included 8 single nucleotide polymorphisms (SNPs): IL6 rs1800795, VDR rs731236, KCNJ11 rs5219, ADRB2 rs1042713, ADRB2 rs1042714, TRHR rs16892496, MSTN rs1805086, UCP3 rs1800849. **Results**: In martial artists, the main predictors were genes responsible for adrenoreceptor sensitivity (ADRB2) and neuroimmune interactions (IL6). In hockey players, the most significant predictors were genes involved in muscle growth (MSTN), energy metabolism (UCP3), and neuroendocrine regulation (TRHR). These findings indicate that similar resting HRV parameters in athletes from different sports may be associated with different genetic polymorphisms, reflecting sport-specific physiological adaptations to training loads. **Conclusions**: The results highlight the sport-specific nature of genetic determinants of autonomic regulation. In martial artists, genes related to the immuno-adrenergic axis (IL6, ADRB2) appear to play a dominant role, whereas in hockey players neuroendocrine, muscle-metabolic, and mitochondrial factors (TRHR, MSTN, UCP3) demonstrate greater influence. The observed interactions between genotypes and FEV_1_ emphasize the importance of transitioning from generalized approaches toward personalized monitoring strategies in sports science.

## 1. Introduction

The aim of this pilot study was to evaluate the hierarchical contribution of individual genetic polymorphisms to the variability of autonomic regulation parameters and respiratory function in athletes of different sport specializations using Classification and Regression Tree (CRT) analysis.

The autonomic nervous system (ANS) represents one of the key mechanisms underlying physiological adaptation in athletes. The balance between the sympathetic (SNS) and parasympathetic (PNS) divisions of the ANS regulates cardiovascular and respiratory responses to physical exertion and significantly influences recovery processes and long-term functional adaptation to systematic training [1,2]. These mechanisms are complex and remain insufficiently understood. Several authors suggest that genetic determinants shape the personal pattern of ANS responses to physical load.

Physical activity, through immunometabolic reprogramming, increases vagal tone, reduces sympathetic activity, and modulates the balance between pro-inflammatory and anti-inflammatory cytokines, particularly the IL-6/IL-10 ratio [3]. Data on the effect of IL6 gene polymorphism (primarily rs1800795, –174 G/C) on circulating interleukin-6 levels and inflammatory responses are contradictory. Carriers of the G allele are more frequently associated with increased IL-6 production and a pro-inflammatory phenotype, which in some studies has been linked to advantages in strength/sprint disciplines, while the C allele has been associated with higher IL-6 levels under certain conditions or with an increased risk of muscle damage [3,4,5]. These discrepancies appear to depend on the population characteristics, exercise modality, and context (acute versus chronic training).

Beyond IL-6-mediated inflammatory mechanisms, ANS function may also be influenced by genetic factors involved in neuroendocrine regulation, skeletal muscle physiology, and mitochondrial energy metabolism. Collectively, these mechanisms determine complex physiological adaptation and may contribute to the formation of personal autonomic profiles in athletes. In this context, polymorphisms of the TRHR, MSTN, and UCP3 genes are of particular interest. The TRHR gene participates in the regulation of the hypothalamic-pituitary-thyroid axis and systemic metabolic control [6]. Thyroid hormone signaling plays an important role in heart rate regulation through both intrinsic cardiac mechanisms and autonomic pathways governing sympathetic–parasympathetic balance [7]. Consequently, genetic variability in the TRHR gene may indirectly modulate ANS activity. The MSTN (myostatin) gene regulates skeletal muscle growth and differentiation, thereby influencing muscle efficiency and cardiorespiratory adaptation [8]. The potential relationship between myostatin signaling and autonomic regulation remains insufficiently clarified. In the study by Lim et al. (2018), the authors noted that the influence of myostatin on ANS regulation remains inconclusive [9]. Genes involved in mitochondrial energy metabolism were also included due to their potential influence on autonomic regulation. The UCP3 gene is involved in mitochondrial uncoupling of oxidative phosphorylation in skeletal muscle, potentially modulating energy efficiency during exercise. Importantly, previous investigations in trained athletes demonstrated an association between the UCP3-55C/T polymorphism and heart rate variability (HRV) parameters. Carriers of the T allele exhibited higher parasympathetic HRV indices compared with individuals possessing the C/C genotype [10]. However, evidence regarding the direct impact of polymorphisms in these genes on ANS parameters (including HRV) in athletes remains limited and warrants further investigation.

The VDR, KCNJ11 (formerly referred to as KCNJ1), and ADRB2 genes may affect ANS function through mechanisms related to neuroendocrine regulation, ion transport, and adrenergic signaling. The rs731236 (TaqI) polymorphism of the VDR gene has been associated with an increased risk of cardiovascular alterations via cardiac remodeling. The mutant G allele (designated as «t» in some classifications) has been linked to approximately a 19% increased risk compared with A (T) allele, although these findings have primarily been reported in the White population [11]. The rs5219 (E23K) polymorphism of the KCNJ11 gene, particularly in T allele carriers, may contribute to improved cardiopulmonary function following high-intensity training [12]. The rs1042713 (Gly16Arg) and rs1042714 (Gln27Glu) polymorphisms of the ADRB2 gene are among the most extensively studied in elite athletes: in several studies, carriers of the G (Glu27) allele demonstrated increased low-frequency (LF) components of HRV; however, these findings have not been consistently replicated [13,14,15,16,17].

In the present study, eight key polymorphic variants of aforementioned genes were selected based on their potential influence on ANS function, HRV, forced expiratory volume in one second (FEV_1_), and overall adaptation of elite athletes to physical exertion. Given the contradictory or limited evidence, particularly in elite athletes, we assumed that the investigated genotypes would be associated with various changes in HRV and FEV_1_ parameters. The findings are expected to expand current understanding of the genetic architecture of exercise adaptation in elite athletes and contribute to the development of personalized training strategies based on genetic profiling.

The specific gene polymorphisms analyzed in this study were IL6 rs1800795, VDR rs731236, KCNJ11 rs5219, ADRB2 rs1042713, ADRB2 rs1042714, TRHR rs16892496, MSTN rs1805086, and UCP3 rs1800849, selected due to their presumed associations with physical performance and endurance.

## 2. Materials and Methods

Study design: An exploratory pilot study was conducted. The study included male elite athletes aged 20–30 years who voluntarily provided written informed consent to participate.

Inclusion criteria: regular professional training and competitive activity, documented athletic achievements and participation in national and international competitions were required, with sport representing the primary professional activity. Athletes held official qualifications corresponding to the titles Master of Sport or International Master of Sport, had been engaged in regular training under professional coaching supervision for more than two years, and maintained elite athlete status for a period ranging from 2 to 6 years.

Exclusion criteria: acute and chronic diseases, injuries, use of medications affecting cardiovascular and respiratory function, participation in competitions or the post-competition recovery period and refusal to participate in the study.

A total of 91 athletes were included. All participants belonged to an ethnically homogeneous population. The study protocol complied with the ethical principles of the Declaration of Helsinki and was approved by the Bioethics Committee of Karaganda Medical University (Protocol No. 15, 17 November 2023). The study was conducted during the off-season period, when no competitive events or intensive training loads were present and outside the recovery period. The athletes were divided into two groups: hockey players (*n* = 48) and combat sport athletes (*n* = 43). The mean body volume (1) of the athletes was calculated using the following equation:

(1) $$V=\frac{m}{\rho }$$

where m represents body mass, and ρ denotes mean body density.

The mean body volume in combat sport athletes was 82.66 ± 8.9 L, whereas in hockey players it was 78.79 ± 9.6 L. Each participant underwent genetic testing for eight SNPs, and HRV (LF, HF) as well as spirometric parameters (FEV_1_) were assessed. The molecular genetic analysis included 8 SNPs: IL6 rs1800795, VDR rs731236, KCNJ11 rs5219, ADRB2 rs1042713, ADRB2 rs1042714, TRHR rs16892496, MSTN rs1805086, UCP3 rs1800849.

Genetic analysis. Biological sample collection and genotyping were performed at the accredited molecular genetics laboratory TreeGene (Almaty, Kazakhstan). The laboratory complies with the requirements of ST RK ISO 15189-2015 [18] (the national equivalent of the international standard ISO 15189:2022 (https://www.iso.org/standard/76677.html?hl=ru-RU (accessed on 10 March 2026)) for medical laboratories), confirmed by accreditation and annual external quality assessment (EQA/PT) participation in international programs including GEDNAP and RFB, and others, as indicated on the laboratory’s official website).

Buccal epithelial cells were collected using a non-invasive sterile buccal swab method. Genotyping of the selected polymorphisms was performed using polymerase chain reaction (PCR) with full quality control procedures, including validated protocols, positive and negative controls, no-template controls, and assessment of repeatability and reproducibility according to accreditation standards.

All pre-analytical, analytical, and post-analytical procedures were performed by qualified personnel using certified equipment and reagents ensuring high analytical reliability (sensitivity and specificity > 95–99%, depending on the SNP). The genotyping success rate was 100%.

HRV assessment. Electrocardiograms were recorded using a Cardipia 406N device (USA) in 2 standard leads for 10 min, HRV analysis was performed using Kubios HRV Standard version 2.2 computer program (Kubios, Finland, (Kubios, www.kubios.com, accessed on 20 February 2026)). To record the cardiogram, we tried to select rooms with the same characteristics and comfortable conditions: air temperature +23 °C, relative humidity 50%, and absence of external noise or odors. Since temperature, humidity, and atmospheric pressure may influence HRV measurements [19,20,21], all recordings were performed in the morning and within the same seasonal period (off-season). Participants were instructed to abstain from caffeine, nicotine, alcohol, sexual activity, and intense physical activity the day before and on the day of testing. Adequate sleep was required, and no pharmacological agents or sports supplements were permitted for at least four weeks prior to the examination. Before ECG recording, participants rested for 15 min in a dimly lit room while seated in a chair with armrests. Given the identical conditions, we assumed that the influence of the external environment was minimal. Before the study, the subjects were asked again about their well-being. During the ECG, medical personnel asked the subjects not to move; if there was any movement or coughing, the ECG was temporarily stopped. All artifacts were excluded from the ECG in manual mode.

Spirometry. Immediately after HRV assessment, spirometry was performed using a calibrated BTL-08 Spiro Pro spirometer (Ashford, Kent, UK) in accordance with ERS recommendations (GLI-2012). Testing was conducted in the seated position with three technically acceptable respiratory maneuvers. The attempt with the highest values was selected for analysis. Parameters were analyzed both as absolute values and as percentages of predicted values, taking into account gender, age, height, and ethnicity. Since population-specific spirometric reference equations for the Kazakh population have not been published, spirometry interpretation in the present study was performed using the Global Lung Function Initiative reference equations (GLI-2012) for the European population. This methodological approach has previously been used in studies conducted in Kazakhstan due to the absence of validated national reference values [22].

Statistical analysis. Statistical analysis was performed using descriptive statistics (median, interquartile range, minimum–maximum). The level of statistical significance was set at *p* < 0.05. Normality of distribution was assessed using the Shapiro–Wilk test. Due to non-normal distribution, group comparisons were performed using non-parametric methods, specifically the Kruskal–Wallis test followed by Dunn’s post hoc test (with Bonferroni correction).

Hardy–Weinberg equilibrium (HWE) was assessed separately for each SNP in both study groups using the chi-square test as part of genotype quality control procedures. Genotyping completeness was high across all analyzed loci, and genotype data were obtained for all study participants. Predictors of HRV parameters were identified using CRT analysis. Dependent variables included HRV frequency-domain indices: high-frequency (HF) and low-frequency (LF) power. Independent predictors included FEV_1_ and genetic polymorphisms of selected genes (categorical variables). Separate trees were constructed separately for each dependent variable. For continuous outcome variables, node splitting was based on minimization of the residual sum of squares (RSS). Tree complexity was controlled using cost–complexity pruning with internal cross-validation to reduce the risk of overfitting and to determine the optimal tree size. Owing to the relatively small sample size, the dataset was not divided into separate training and testing subsets. Model performance was evaluated using the improvement index provided by the CRT procedure in SPSS (IBM SPSS Statistics 27.0.1), which reflects the proportional reduction in prediction error relative to the null model. This metric was interpreted as an analogue of the coefficient of determination (R^2^) and was used as a descriptive measure of explained variance for all regression tree models within the study sample. To further reduce overfitting and assess model stability, internal validation was performed using 10-fold cross-validation within the CRT procedure. Because SPSS provides cross-validated risk estimates rather than cross-validated R^2^ values, model robustness was evaluated by comparing risk estimates calculated on the original dataset (resubstitution risk) with risk estimates obtained through cross-validation.

All genotype categories were retained in the analysis. During CRT model construction, genotype groups were automatically combined by the algorithm into binary partitions that maximized node homogeneity according to the splitting criterion. Consequently, rare genotypes were not excluded but were grouped with the most statistically similar category during tree formation.

The study was conducted within the framework of a grant from the Ministry of Education and Science of the Republic of Kazakhstan 0124RK00898 AP23490397 titled “Analysis of molecular and genetic mechanisms of endurance in athletes with effective sports performance” (2024–2026) at Karaganda Medical University during 2024–2025.

## 3. Results

### 3.1. Genetic Characteristics

The overall genotype distribution across athlete groups is presented in Table 1. Deviation from Hardy–Weinberg equilibrium was observed for the ADRB2 polymorphisms rs1042713 and rs1042714 exclusively in the combat sport athlete group. Since genotype data were available for all participants and allele frequencies generally fell within expected ranges, these deviations were unlikely to be attributable to genotyping errors.

Deviations from HWE were observed for ADRB2 polymorphisms in the combat sport athlete group.

The genotype frequencies and HRV/spirometry parameters in athletes are presented in Table A1 and Table A2.

### 3.2. Predictive Models for Combat Sport Athletes

**Figure 1:**
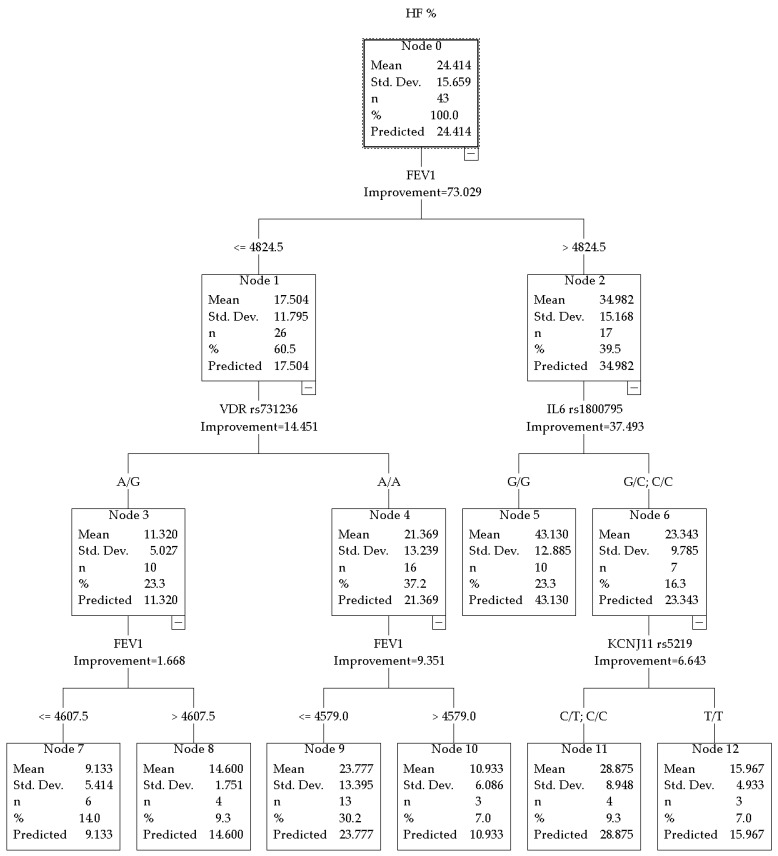
CRT model for HF% in combat sport athletes.

The CRT model for HF% in combat sport athletes demonstrated high predictive power with a total R^2^ = 0.730. The hierarchy of predictors is presented in Figure 1.

When divided into nodes, the distinguishing factor was the absolute FEV_1_ value, with a threshold of 4824.5 mL.

In the subgroup of combat sport athletes with FEV_1_ above the threshold, further branching according to HF% was associated with the IL6 rs1800795 polymorphism. 23.3% of the total sample—athletes with the G/G genotype (*n* = 10)—demonstrated the highest mean resting HF% values, equal to 43.130 ± 12.685. Carriers of the C allele (G/C or C/C genotypes, *n* = 7) had lower values: HF% = 23.343 ± 9.785.

In the subgroup with FEV_1_ below the threshold, the VDR rs731236 polymorphism was identified as a significant predictor of HF%. Athletes with the A/A genotype of the VDR gene (*n* = 13) showed a mean HF% of 23.777 ± 13.395. On the other hand, heterozygous A/G carriers (*n* = 10) demonstrated the lowest values, where HF% = 11.320 ± 5.027.

Additional stratification within the subgroup characterized by higher FEV_1_ values and the C allele of IL6 gene was associated with the KCNJ11 rs5219 polymorphism.

**Figure 2:**
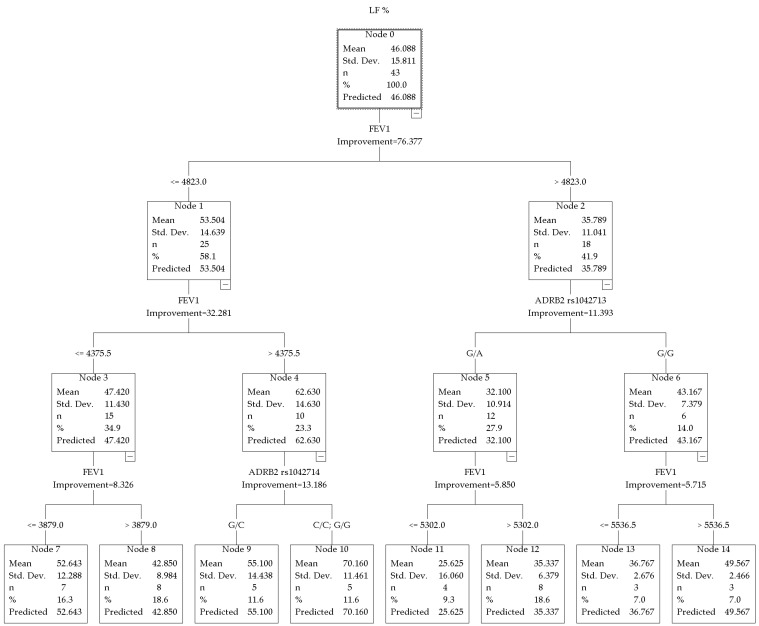
CRT model for LF% in combat sport athletes.

The model for LF% also had high predictive power with a total R^2^ = 0.763 and revealed a different hierarchy of predictors (Figure 2).

A key finding in this study was the identification of the ADRB2 rs1042714 polymorphism within the subgroup exhibiting the highest LF% values (node 4). This genetic marker divided the group into two categories, with different levels of sympathetic activity. The first was the heterozygous G/C genotype (node 9, *n* = 5), where the average LF% was 55.100 ± 14.438. The second was the homozygous genotypes C/C and G/G (node 10, *n* = 5), with the highest LF% in the tree, equal to 70.160 ± 11.461.

Athletes with FEV_1_ ≤ 4823 mL (node 1, *n* = 25) demonstrated higher resting LF% values (53.504 ± 14.639) compared with athletes with FEV_1_ > 4823 mL (node 2, *n* = 18; 35.789 ± 11.041).

Further stratification within the FEV_1_ ≤ 4823 mL group was determined by an additional FEV_1_ threshold. Athletes with FEV_1_ ≤ 4375.5 mL (node 3, *n* = 16) had LF% = 47.420 ± 11.430. Athletes with FEV_1_ in the range of 4376–4823 mL (node 4, *n* = 10) demonstrated the highest values in this branch, LF% = 62.630 ± 14.630.

### 3.3. Predictive Models for Hockey Players

**Figure 3:**
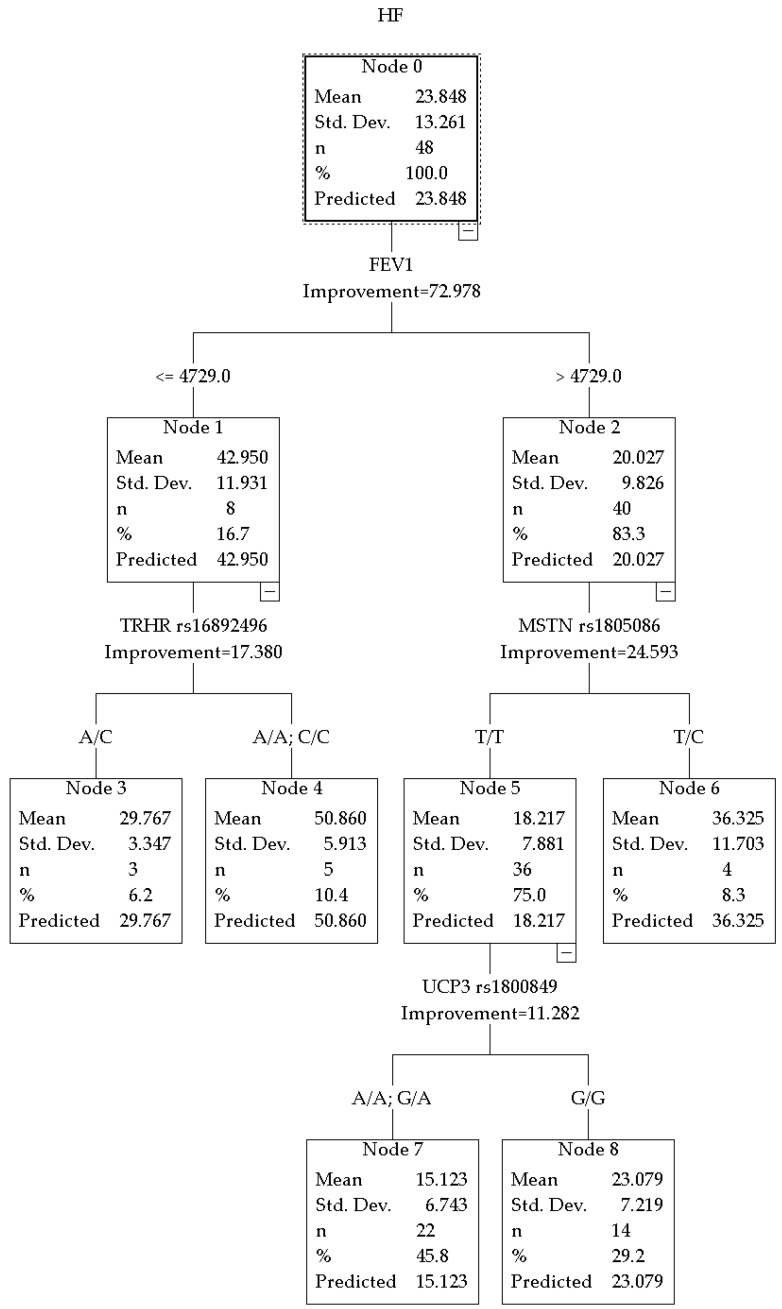
CRT model for HF% in hockey players.

The overall predictive performance of the CRT model for HF% of the autonomic regulation in hockey players was R^2^ = 0.729 (improvement = 72.978), revealing a hierarchy of predictors different from that observed in combat sport athletes (Figure 3).

In the subgroup with FEV_1_ ≤ 4729 mL, the key predictor was the TRHR rs16892496 polymorphism. The genetic marker divided this subgroup into two categories. Carriers of the A/C genotype (node 3, *n* = 3) had an average HF% = 29.767 ± 3.347. Homozygous A/A and C/C carriers (node 4, *n* = 5) had the highest value in the tree, HF% = 50.860 ± 5.913.

In the subgroup with FEV_1_ > 4729 mL, further stratification was determined by the MSTN rs1805086 polymorphism. Carriers of the T/T genotype (node 5, *n* = 36) had an average value of 18.217 ± 7.881. Carriers of the T/C genotype (node 6, *n* = 4) had a mean value of HF% = 36.325 ± 11.703.

Within the subgroup with low HF% values (node 5), additional splitting was associated with the UCP3 rs1800849 polymorphism. Carriers of the A allele (A/A; G/A genotypes) (node 7, *n* = 22) demonstrated the lowest values in the sample HF% = 15.123 ± 6.743. Carriers of the G/G genotype (node 8, *n* = 14) had higher values of HF% = 23.079 ± 7.219.

**Figure 4:**
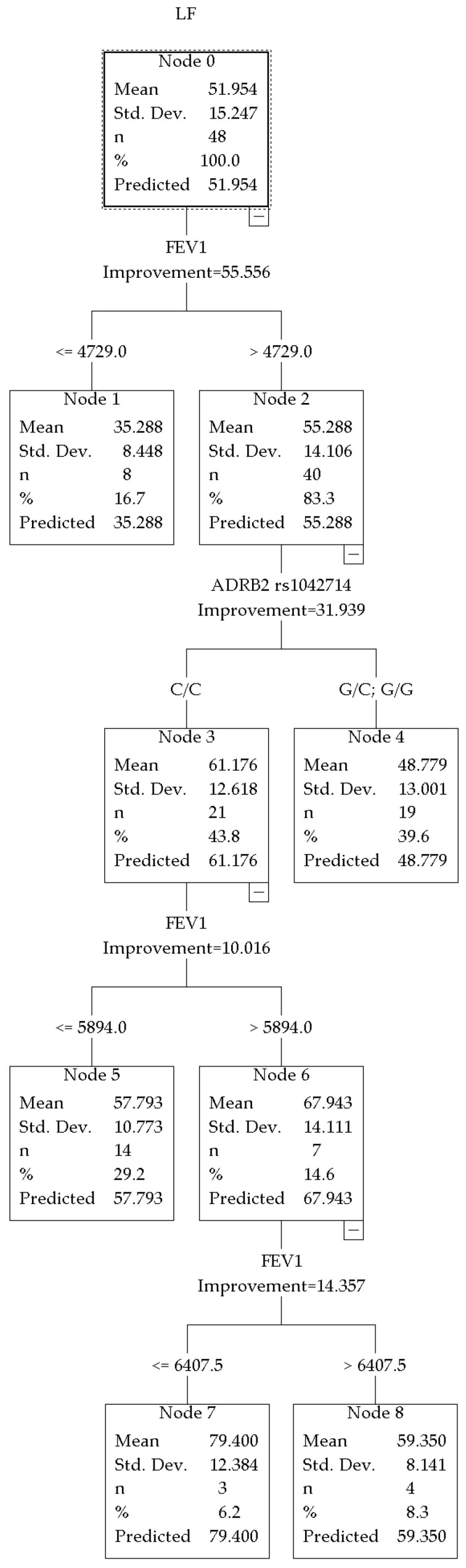
CRT model for LF% in hockey players.

The overall predictive power of the model for LF% in hockey players was R^2^ = 0.55 (improvement = 55.556) (Figure 4). The primary splitting variable was FEV_1_, with a threshold of 4729 mL.

In the subgroup with high LF% (node 2), the key predictor was the ADRB2 rs1042714 polymorphism (encoding the β_2_-adrenergic receptor). The genetic marker divided the subgroup into two categories, with different levels of sympathetic activity. The first was homozygous C/C genotypes (node 3, *n* = 21), where the average LF% was 61.176 ± 12.618. The second category consisted of heterozygous genotypes G/C and G/G (node 4, *n* = 19), where the average LF% was 48.779 ± 13.001.

The initial split of the model was determined by FEV_1_. The athletes were divided into two groups. The first group had a lower LF% (node 1, *n* = 8; average 35.288 ± 8.448). The second group had a higher LF% (node 2, *n* = 40; average 55.288 ± 14.106).

Within the C/C homozygous subgroup (node 3), further stratification was determined by FEV_1_. Athletes with relatively higher lung volume indicators (node 6, *n* = 7) demonstrated even higher LF% = 67.943 ± 14.111, whereas hockey players with slightly lower FEV_1_ (node 5, *n* = 14) showed LF% = 57.793 ± 10.773. Further splitting within node 6 (by FEV_1_ threshold) allowed us to identify a cohort of athletes with the highest LF% = 79.400 ± 12.384 values in the entire sample (node 7, *n* = 3).

No genetic markers were identified in the group with initially low LF% values (node 1).

Comparison of risk estimates calculated on the original dataset (resubstitution risk) and those obtained through cross-validation demonstrated comparable values across all HF% and LF% models in both combat sport athletes and hockey players, indicating model stability and the absence of substantial overfitting (Table 2).

Additionally in Appendix section, a summary table comparing key predictors across all four CRT models has been added to facilitate comparison (Table A3).

## 4. Discussion

Although the study was conducted on a relatively limited sample size (48 hockey players and 43 combat sport athletes), multilevel decision tree models incorporating 8 SNPs together with continuous physiological variables were constructed. While some terminal nodes contained only 3–5 participants, the models revealed potentially informative patterns and associations. As a result of applying the CRT method to analyze HRV in elite combat sport athletes (*n* = 43), the first split at the FEV_1_ threshold of 4824.5 mL explained the largest proportion of variation in the HF% component. Athletes with high FEV_1_ (>4824.5 mL) demonstrated a mean HF value of 34.982%, whereas in those with lower FEV_1_ (≤4824.5 mL) the value was only 17.504%. The average HF% for the entire sample (24.414%) was lower than typical values reported for well-adapted elite endurance athletes, where HF% at rest often reaches 40–60% and above, reflecting pronounced parasympathetic tone [23]. Thus, the observed average of 24.4% may indicate the relative dominance of the SNS even at rest and between training sessions in some athletes. It is currently difficult to assess this condition as an adaptive process to training or as insufficient recovery, overwork/overtraining due to the lack of large-scale studies taking into account various factors [24].

In nodes 5 and 6 of the decision tree, further branching according to the IL6 rs1800795 (-174C) polymorphism showed that athletes with high FEV_1_ (>4824.5 mL) carrying the G/G genotype had significantly higher HF% values at rest (43.130 ± 12.685%) compared with carriers of the C allele (G/C or C/C genotypes; 23.343 ± 9.785%). Thus, the G/G genotype appears to have a greater potential for parasympathetic activity. Although the G/G genotype is traditionally associated with higher IL6 promoter transcriptional activity of the IL6 promoter and increased IL-6 levels in response to various stimuli [4], in the context of chronic adaptation to physical exercise, it may exert a protective effect, including reduced circulating creatine kinase levels, decreased muscle damage, faster tissue repair, and enhanced anti-inflammatory mechanisms (stimulation of IL-1ra, IL-10, suppression of TNF-α and IL-1β) [3,25]. Cooper TM et al. (2014) reported an inverse relationship between inflammatory markers, including IL-6, and HF power, suggesting a protective role of the PNS in limiting or preventing excessive inflammatory responses [26]. Individuals carrying one or more IL-6-174C alleles demonstrated higher peak creatine kinase (CK) levels after exercise compared with individuals homozygous for the G allele. In addition, carriers of the C allele have been reported in some studies to have an increased risk of chronic obstructive pulmonary disease (COPD) and a more rapid decline in FEV_1_ [27,28], which was confirmed by a 2019 meta-analysis (for the C/C genotype OR = 1.31, 95% CI 1.04–1.64, *p* = 0.01) [29]. Thus, the literature data are consistent with our findings: carriers of the G/G genotype with high FEV_1_ demonstrate the highest parasympathetic tone (HF% = 43.13%). Taken together, these findings may indicate that the IL6 rs1800795 polymorphism could be involved in autonomic regulation in athletes with high respiratory capacity. One biologically plausible explanation involves interactions between inflammatory signaling and vagal modulation described within the framework of the cholinergic anti-inflammatory pathway. To the best of our knowledge, no mechanisms explaining the observed findings have been described in the available literature. Therefore, the associations identified in the present study should be considered as hypotheses for future research rather than as definitively established relationships.

Analysis of nodes 3 and 4 showed that FEV_1_ values were initially lower in athletes carrying the VDR rs731236 polymorphism. The vitamin D receptor gene is known to be involved in the regulation of the immune response, particularly influencing suppressor mechanisms and susceptibility to inflammatory and autoimmune processes [30]. The AA genotype of the VDR gene may be associated with more favorable conditions for maintaining parasympathetic activity. One possible explanation involves the regulatory role of VDR in the renin–angiotensin system, which may thereby influence vascular tone and blood pressure regulation [31]. Since the renin–angiotensin system is closely interconnected with ANS activity [32,33], VDR-related signaling could potentially contribute to autonomic balance modulation. However, these mechanisms remain hypothetical and require further experimental confirmation. Our results suggest that carriers of the A/A genotype demonstrate higher HF% values than A/G heterozygotes, with an almost twofold difference (23.8% vs. 11.3%). It is likely that the A/A variant of the VDR gene creates more favorable conditions for maintaining parasympathetic activity, possibly through its involvement in the regulation of the renin-angiotensin system and the associated ANS tone. In heterozygotes, this mechanism appears to manifest as lower parasympathetic tone in the presence of initially lower pulmonary function parameters.

In a relatively small subgroup of combat sport athletes (*n* = 7) with high baseline FEV_1_ (>4824.5 mL) carrying the KCNJ11 rs5219 (E23K) polymorphism, carriers of the C allele (C/C or C/T genotypes) demonstrated higher HF% values at rest (≈28.875%) compared with T/T homozygotes (≈15.967%) (nodes 11, 12). This finding indicates potentially more pronounced parasympathetic tone and a more favorable recovery profile in C-allele carriers within the context of FEV_1_ values. In one study of healthy young individuals following 12 weeks of high-intensity interval training (HIIT), the rs5219 T allele was associated with a greater increase in VO_2_max (maximal oxygen consumption), suggesting an advantage of T-allele carriers in improving aerobic capacity (*p* = 0.015) [12]. However, in our study the opposite pattern was observed: the C allele was associated with higher HF% values at rest.

When analyzing resting LF% in combat sport athletes, particular attention was drawn to the ADRB2 rs1042714 polymorphism (Gln27Glu; a C > G substitution resulting in the replacement of glutamine with glutamic acid at position 27 of the β_2_-adrenoceptor protein). In our study, the highest resting LF% values (70.160 ± 11.461%) were observed in carriers of homozygous genotypes (C/C and G/G; node 10), whereas G/C heterozygotes (node 9) demonstrated lower values (55.100 ± 14.438%). This effect was not observed in the entire sample, but only in the subgroup with FEV_1_ in the range of 4376–4823 mL (node 4), where the baseline LF% was already high (62.630 ± 14.630%). The present findings suggest that homozygosity for rs1042714 regardless of allele type, is associated with more pronounced sympathetic tone compared with the heterozygous state within this specific FEV_1_ range.

The literature provides no clear consensus regarding the association between rs1042714 and LF components of HRV: Matsunaga et al. (2007) reported that carriers of the Glu27 (G) allele demonstrated higher LF power compared with Gln27 (C/C) homozygotes in healthy Japanese men [16]. In another study, athletes carrying the Glu27 allele showed increased systolic blood pressure in response to cold stress, indirectly reflecting enhanced sympathetic reactivity [34]. However, several studies have reported no direct association between rs1042714 and HRV parameters or resting heart rate [34]. The present findings differ from some previous observations, which may be explained by interaction with FEV_1_ and the specific characteristics of the combat sport population.

Summarizing the data on HF% and LF%, two genetic markers associated with autonomic regulation at rest in combat sport athletes can be identified. The parasympathetic component (HF%) was associated with IL6 rs1800795 polymorphism. The sympathetic component (LF%) was associated with the ADRB2 rs1042714 polymorphism. Notably, these genes appear to exert their effects in different FEV_1_ ranges. IL6 was significant in the cohort with FEV_1_ values above 4824.5 mL, whereas ADRB2 was significant in the cohort with FEV_1_ values in the range of 4376–4823 mL.

The results obtained in the sample of hockey players substantially expand the understanding of the genetic determinants of autonomic balance in athletes involved in interval sports. Unlike combat sport athletes, in whom the IL6 and ADRB2 genes played a leading role, in hockey players the main contribution appears to be associated with polymorphisms related to neuroendocrine regulation (TRHR), muscle mass and metabolism (MSTN), and mitochondrial energy metabolism (UCP3).

Interestingly, among hockey players with FEV_1_ values below the threshold (≤4729 mL) (Figure 3), the TRHR rs16892496 polymorphism contributed to parasympathetic activity. To the best of our knowledge, such an association has not previously been reported in the literature. Thyrotropin-releasing hormone (TRH) and its receptor (TRHR) are known to influence the cardiovascular system, autonomic regulation, and metabolism through thyroid hormones [6]. In our study, a substantial difference in HF% was observed between genotypes: 50.860% (node 4) versus 29.767% (node 3). Considering that this effect was observed only at lower FEV_1_ values, a compensatory increase in parasympathetic activity in a specific TRHR genotype under conditions of reduced pulmonary function can be assumed. This mechanism may contribute to maintaining autonomic balance during regular high training loads.

In hockey players with high FEV_1_ (>4729 mL), the association between the MSTN rs1805086 polymorphism and parasympathetic activity is noteworthy: carriers of the heterozygous T/C genotype demonstrated HF% values of 36.325% (node 6), whereas T/T homozygotes showed nearly twofold lower values (18.217%, node 5). Myostatin is known as a negative regulator of skeletal muscle growth [8], but it is also expressed in the myocardium and vascular smooth muscle [35]. Therefore, MSTN variants may influence not only muscle mass, but also the ANS and respiratory musculature (indirectly, through respiratory muscle strength). Higher parasympathetic tone in T/C carriers may contribute to more effective recovery and stronger vagal influence following physical loads.

Among hockey players with initially low parasympathetic activity (node 5, mean HF% = 18.217%), additional stratification by the UCP3 rs1800849 polymorphism revealed genotype-dependent differences: carriers of the A allele (A/A and G/A, node 7) demonstrated the lowest HF% values in the sample (15.123%), whereas G/G homozygotes (node 8) showed moderately higher values (23.079%). The UCP3 protein is expressed predominantly in skeletal muscles and participates in the regulation of oxidative stress and fatty acid metabolism [36]. It should also be taken into account that the UCP3 rs1800849 polymorphism may be reported using different allelic notations (A/G or C/T) depending on the DNA strand used for annotation. It should be noted that the same locus may be designated differently depending on the DNA strand; thus, what is described as T/C in one study may appear as A/G in another. UCP3 polymorphisms have also been associated with HRV: in several studies, the T/T genotype has been linked to higher HRV and higher parasympathetic activity [37,38]. The present findings generally align with these observations, suggesting a possible association between the A allele and lower parasympathetic modulation. One biologically plausible explanation may involve mitochondrial metabolic regulation and oxidative stress pathways, which have been proposed to influence autonomic cardiovascular control. The obtained results should be regarded as preliminary (exploratory findings) and require external cross-validation in independent cohorts, preferably multicenter samples with a larger number of participants.

When analyzing LF% indicators at rest in hockey players (Figure 4), the association between the rs1042714 polymorphism of the ADRB2 gene (Gln27Glu) and sympathetic activity is of particular interest. The highest LF% values were observed in carriers of the homozygous C/C genotype (node 3, mean 51.161 ± 12.618%), whereas carriers of the G allele (genotypes G/C and G/G, node 4) demonstrated lower sympathetic tone (mean 48.779 ± 13.001%). Moreover, in C/C carriers, higher FEV_1_ values (>5894 mL) were accompanied by even more pronounced sympathetic activity (LF% up to 67.943% in node 6). These findings are consistent with the results obtained in combat sport athletes (Figure 2). Thus, the rs1042714 polymorphism may be considered one of the genetic factors associated with the level of sympathetic activity at rest in hockey players. However, its contribution is not realized in isolation, but in close interaction with pulmonary function indicators (FEV_1_): the first split of the decision tree by the FEV_1_ threshold of approximately 4729 mL explained the largest proportion of LF% variability, after which the ADRB2 polymorphism became the determining factor in the high FEV_1_ branch. These findings emphasize the context-dependent nature of the influence of rs1042714 on the autonomic profile and highlight the importance of considering physiological factors (lung function) when interpreting HRV in athletic populations, particularly in sports characterized by high sympathetic mobilization.

The present study had a pilot exploratory design. Its uniqueness lies in the fact that, when comparing the results obtained in the two samples (combat sport athletes and hockey players), it becomes evident that the set of genetic markers associated with resting autonomic regulation differs between the groups. In combat sport athletes, the main predictors were genes responsible for adrenoreceptor sensitivity (ADRB2) and neuroimmune interactions (IL6). The specificity of combat sports is associated with high psycho-emotional stress and the need for rapid mobilization and recovery. Under such conditions, regulation at the receptor and immune levels becomes particularly important. In hockey players, other genes became significant—those involved in muscle growth regulation (MSTN), energy metabolism (UCP3), and neuroendocrine control (TRHR). Anaerobic power, maintenance of muscle mass, and metabolic efficiency are crucial for hockey performance. Therefore, the resting autonomic profile of these athletes appears to be associated primarily with genes determining metabolic and structural characteristics of muscle tissue. Thus, the same HRV indicators at rest in athletes from different sports may be associated with different genetic polymorphisms. This likely reflects sport-specific patterns of adaptation to stress in combat sport athletes and hockey players. In both cases, these genes regulate autonomic tone, but through different physiological mechanisms. The obtained data allowed the identification of several potentially important associations that have not previously been described in the literature for this specific professional group. Due to the limited sample size and its regional character, external cross-validation in independent cohorts is required to confirm the observed associations. Until such studies are conducted, the presented conclusions should be interpreted as hypothesis-generating rather than as definitive evidence of established relationships.

The present study has several limitations that should be considered when interpreting the findings. The relatively small sample size, which is typical for genetic studies involving elite athletes, limits statistical power and may reduce the stability of terminal nodes in CRT models, where some nodes included only 3–5 participants. Therefore, the identified associations should be regarded as exploratory and hypothesis-generating rather than definitive predictive relationships and require confirmation in larger independent cohorts.

Although CRT analysis is relatively robust when applied to small datasets and was used to identify hierarchical genotype–phenotype relationships, the dataset was not divided into independent training and testing subsets due to sample size constraints. Instead, model complexity was controlled using internal cross-validation combined with cost–complexity pruning. Consequently, the present findings should be interpreted as sample-specific and cannot be generalized to external populations. Larger studies incorporating independent validation cohorts are therefore required.

The cross-sectional design of the study does not allow causal relationships to be established between genetic polymorphisms, autonomic regulation parameters, and respiratory function. Accordingly, the observed associations represent statistical relationships that should be verified in prospective longitudinal investigations.

Because multiple CRT models were constructed using the same set of predictors, the possibility of chance associations cannot be completely excluded. Thus, the identified hierarchical predictor structures should be interpreted as exploratory findings requiring replication in independent samples.

In accordance with the study objectives, several potential confounding variables were not included in the analyses, including age, training volume, training experience, training programs, body mass index, fat and lean mass distribution, nutritional status, and macro- and micronutrient intake, all of which may influence HRV parameters and FEV_1_. No adjustments or stratification for these variables were performed. Their potential contribution should be addressed in future large-scale cohort studies. Training load, athletic experience, body composition, and nutritional factors were not incorporated into the models and should be considered in future investigations involving larger samples.

Since genotype data were obtained for all participants and allele frequencies remained within expected ranges, deviations from Hardy–Weinberg equilibrium observed for ADRB2 polymorphisms in one study group are unlikely to reflect genotyping errors. Rather, these deviations may be attributable to the relatively small sample size or to other biological or population-specific factors that cannot currently be fully explained due to limited available evidence.

The study employed a limited set of functional assessment methods, including resting HRV recording and spirometry, which restricts interpretation of the physiological mechanisms underlying the observed genotype–phenotype associations. The absence of functional stress testing, ECG parameters, blood pressure measurements, and laboratory biomarkers (e.g., interleukins, thyroid hormones, vitamin D, and catecholamines) limits the ability to provide a comprehensive explanation of autonomic regulatory patterns in athletes.

Finally, interpretation of the relatively low HF% values observed in both athlete groups compared with those typically reported in well-adapted endurance-trained elite populations remains challenging. It remains unclear whether the predominance of sympathetic modulation at rest represents a physiological adaptation to intermittent high-intensity training and sport-specific demands or reflects features of autonomic imbalance. Similarly, the relatively elevated sympathetic tone observed in certain genotype-defined subgroups, particularly ADRB2 rs1042714 homozygotes, may represent adaptive regulatory patterns rather than dysfunction in the absence of clinical abnormalities. These observations highlight the need for integrative studies combining genetic, physiological, and functional markers to enable personalized assessment of ANS adaptation across different sport disciplines.

## 5. Conclusions

The CRT method allowed the identification of a hierarchical structure of determinants of autonomic status in elite athletes (combat sport athletes and hockey players) at rest. The leading physiological factor across all models was pulmonary function (FEV_1_), which determined the first split of the decision tree and modulated the contribution of genetic polymorphisms.

In combat sport athletes, parasympathetic activity (HF%) was most pronounced in the presence of high FEV_1_ combined with the G/G genotype of the IL6 gene (rs1800795), which may reflect optimal recovery potential and resistance to prolonged competitive stress. Sympathetic activity (LF%) was associated with the rs1042714 polymorphism of the ADRB2 gene: homozygous genotypes (C/C and G/G) were linked to the highest LF% values in the subgroup with moderate FEV_1_ (4376–4823 mL), which may represent an adaptive phenotype for explosive efforts and rapid mobilization.

In hockey players, parasympathetic tone at rest was determined by the TRHR rs16892496, MSTN rs1805086, and UCP3 rs1800849 polymorphisms. Sympathetic activity (LF%) was associated with rs1042714 of ADRB2, where the C/C genotype combined with high FEV_1_ was linked to the most pronounced sympathetic tone.

The obtained results highlight sport-specific genetic determinants of autonomic regulation: in combat sport athletes, genes related to the immuno-adrenergic axis (IL6, ADRB2) predominate, whereas in hockey players neuroendocrine, muscle-metabolic, and mitochondrial factors (TRHR, MSTN, UCP3) play a major role. The identified interactions between genotypes and FEV_1_ indicate the need to move beyond universal approaches toward personalized monitoring in sports science.

The integration of genetic testing (IL6, ADRB2, TRHR, MSTN, UCP3, etc.) with HRV and spirometry assessment at rest may improve the prediction of adaptive capacity, risk of overtraining, and recovery efficiency. This approach opens prospects for the development of genetically informed personalized training programs and functional monitoring strategies in intermittent sports.

These findings should be interpreted in the context of a pilot exploratory study involving a limited number of participants, which may partially reduce the robustness and generalizability of the obtained results. Future studies should include dynamic assessments (before/after exercise), large samples, and functional testing in order to confirm causal relationships and evaluate the practical applicability of these findings.

## Figures and Tables

**Table 1:** Distribution of genotypes and corresponding Hardy–Weinberg equilibrium (HWE) *p*-values assessed using chi-square tests in 91 elite athletes, including combat sport athletes (*n* = 43) and hockey players (*n* = 48). M and m denote the major and minor allelic variants of each polymorphism, respectively.

Group of Sportsmen	Gene	rsID	M/m	MM	Mm	mm	*p*-Value
Combat sport athletes	IL6	rs1800795	A/G	4.65%	39.53%	55.81%	0.641
	VDR	rs731236	T/C	0.00%	34.88%	65.12%	0.166
	KCNJ11	rs5219	T/C	18.60%	37.21%	44.19%	0.182
	ADRB2	rs1042713	A/G	4.65%	58.14%	37.21%	0.0487
	ADRB2	rs1042714	G/A	2.33%	58.14%	39.53%	0.0219
Hockey players	ADRB2	rs1042714	G/A	8.33%	41.67%	50.00%	0.954
	TRHR	rs16892496	A/G	14.58%	52.08%	33.33%	0.581
	UCP3	rs1800849	C/T	8.33%	45.83%	45.83%	0.644
	MSTN	rs1805086	C/T	0.00%	8.33%	91.67%	0.763

**Table 2:** Internal validation of CRT models based on comparison of resubstitution and cross-validated risk estimates.

Model	Resubstitution Risk	Cross-Validated Risk	Difference (%)
HF1	239.495	248.352	+3.7%
HF2	172.180	178.769	+3.8%
LF2	227.625	249.403	+9.6%
LF1	245.852	257.833	+4.9%

## Data Availability

The raw data supporting the conclusions of this article will be made available by the authors on request.

## Abbreviations

The following abbreviations are used in this manuscript:

HRV	Heart rate variability
ANS	Autonomic nervous system
SNS	Sympathetic nervous system
PNS	Parasympathetic nervous system
SNP	Single nucleotide polymorphism
LF	Low frequency
HF	High frequency
FEV_1_	Forced expiratory volume in one second
PCR	Polymerase chain reaction
ECG	Electrocardiogram
CRT	Classification and Regression Tree
CK	Creatine kinase
COPD	Chronic obstructive pulmonary disease
HIIT	High-intensity interval training
TRH	Thyrotropin-releasing hormone

## Appendix A

Because HF, LF, and FEV_1_ variables demonstrated a non-normal distribution, non-parametric descriptive statistics were applied. Data are presented as median and interquartile range (IQR, 25th–75th percentiles).

**Table A1:** Genotype frequencies and HRV/spirometry parameters in combat sport athletes.

Combat Sport Athletes		N	Median	Percentiles	
IL6 rs1800795				25	75
**HF, %**	C/C	2	16.15	14.60	17.70
	G/C	17	15.60	9.60	25.60
	G/G	24	28.00	16.35	44.70
**LF, %**	C/C	2	52.00	51.60	52.40
	G/C	17	48.40	37.20	59.90
	G/G	24	39.45	33.60	53.25
**FEV_1_, mL**	C/C	2	4904.00	4181.00	5627.00
	G/C	17	4717.00	4252.00	5331.00
	G/G	24	4732.50	4236.50	5348.50
**VDR rs731236**					
**HF, %**	A/A	28	27.70	16.10	40.75
	A/G	15	15.00	10.85	18.00
**LF, %**	A/A	28	38.60	34.35	49.85
	A/G	15	52.40	47.65	60.85
**FEV_1_, mL**	A/A	28	4753.50	4194.50	5435.00
	A/G	15	4727.00	4217.50	5302.00
**KCNJ11 rs5219**					
**HF, %**	C/C	19	29.40	19.70	42.40
	C/T	16	15.45	5.95	23.65
	T/T	8	18.80	13.45	19.80
**LF, %**	C/C	19	39.20	33.60	51.55
	C/T	16	52.00	39.40	58.80
	T/T	8	44.05	34.60	50.95
**FEV_1_, mL**	C/C	19	4709.00	4437.50	5435.00
	C/T	16	4654.63	4151.50	5077.50
	T/T	8	4591.88	3928.00	5247.00
**ADRB2 rs1042713**					
**HF, %**	A/A	2	12.10	9.60	14.60
	G/A	25	26.90	11.70	44.40
	G/G	16	19.50	15.95	26.15
**LF, %**	A/A	2	65.70	51.60	79.80
	G/A	25	41.90	32.20	54.00
	G/G	16	48.15	37.60	56.50
**FEV_1_, mL**	A/A	2	4291.50	4181.00	4402.00
	G/A	25	4822.00	4307.00	5424.00
	G/G	16	4595.00	4031.00	5266.50
**ADRB2 rs1042714**					
**HF, %**	C/C	17	18.30	9.60	29.10
	G/C	25	25.60	16.30	37.40
	G/G	1			
**LF, %**	C/C	17	42.40	39.20	54.00
	G/C	25	42.80	34.80	55.20
	G/G	1			
**FEV_1_, mL**	C/C	17	4498.00	4252.00	5273.00
	G/C	25	4748.00	4166.00	5424.00
	G/G	1			

All genotype categories were included in CRT modeling; grouping of genotypes in decision trees reflects algorithm-driven binary partitioning.

**Table A2:** Genotype frequencies and HRV/spirometry parameters in hockey players.

Hockey Players		N	Median	Percentiles	
TRHR rs16892496				25	75
**HF, %**	A/A	16	24.60	18.25	39.25
	A/C	25	17.30	11.80	25.90
	C/C	7	27.80	21.95	49.70
**LF, %**	A/A	16	49.50	39.70	58.65
	A/C	25	55.10	45.60	65.20
	C/C	7	39.70	35.50	55.65
**FEV_1_, mL**	A/A	16	5484.50	4840.00	5897.00
	A/C	25	5426.00	4992.00	6037.00
	C/C	7	5322.00	4825.00	5595.00
**MSTN rs1805086**					
**HF, %**	T/C	4	34.30	26.85	45.80
	T/T	44	20.55	13.35	27.50
**LF, %**	T/C	4	42.05	37.40	50.50
	T/T	44	50.35	40.45	65.20
**FEV_1_, mL**	T/C	4	5697.50	5420.50	6674.00
	T/T	44	5343.50	4840.00	5949.50
**UCP3 rs1800849**					
**HF, %**	A/A	4	31.65	22.40	44.50
	G/A	22	13.60	11.50	22.30
	G/G	22	26.30	21.10	37.70
**LF, %**	A/A	4	36.15	29.45	53.30
	G/A	22	55.55	45.60	65.50
	G/G	22	48.50	37.90	58.60
**FEV_1_, mL**	A/A	4	4522.50	4291.50	5649.50
	G/A	22	5552.00	5050.00	6138.00
	G/G	22	5291.50	4818.00	5812.00
**ADRB2 rs1042714**					
**HF, %**	C/C	24	19.15	13.70	26.30
	G/C	20	23.00	12.75	35.15
	G/G	4	32.10	17.75	46.35
**LF, %**	C/C	24	59.85	46.55	67.85
	G/C	20	45.70	37.80	54.00
	G/G	4	44.25	36.30	62.45
**FEV_1_, mL**	C/C	24	5552.00	5180.50	6025.00
	G/C	20	5030.00	4801.50	5949.50
	G/G	4	5287.00	4713.00	6594.00

## Appendix B

Additionally, a summary table comparing key predictors across all four CRT models has been added to facilitate comparison.

**Table A3:** Comparative Characteristics of Hierarchical CRT Models.

	Dependent Variable	Primary Predictor	Key Genetic Moderators
**Combat sport athletes**	HF% (Parasympathetic Activity)	FEV_1_ > 4824.5 mL	IL6 rs1800795 (G/G), KCNJ11 rs5219 (C/T, C/C)
		FEV_1_ ≤ 4824.5 mL	VDR rs731236 (A/G, A/A)
	LF% (Sympathetic Activity)	FEV_1_ > 4823.0 mL	ADRB2 rs1042714 (G/G, G/A)
**Hockey players**	HF% (Parasympathetic Activity)	FEV_1_ ≤ 4729.0 mL	TRHR rs16892496 (A/A, C/C),
		FEV_1_ > 4729.0 mL	MSTN rs1805086 (T/T), UCP3 rs1800849 (A/A, G/A)
	LF% (Sympathetic Activity)	FEV_1_ > 4729.0 mL	ADRB2 rs1042714 (C/C)
